# A stroma‐related lncRNA panel for predicting recurrence and adjuvant chemotherapy benefit in patients with early‐stage colon cancer

**DOI:** 10.1111/jcmm.14999

**Published:** 2020-01-27

**Authors:** Rui Zhou, Huiying Sun, Siting Zheng, Jingwen Zhang, Dongqiang Zeng, Jianhua Wu, Zhenhua Huang, Xiaoxiang Rong, Jianping Bin, Yulin Liao, Min Shi, Wangjun Liao

**Affiliations:** ^1^ Department of Oncology Nanfang Hospital Southern Medical University Guangzhou China; ^2^ Department of Medicine Ultrasonics Nanfang Hospital Southern Medical University Guangzhou China; ^3^ Department of Cardiology Nanfang Hospital Southern Medical University Guangzhou China

**Keywords:** chemotherapy responsiveness, colon cancer, prognosis, stroma‐related lncRNA signature

## Abstract

The heterogeneity in prognoses and chemotherapeutic responses of colon cancer patients with similar clinical features emphasized the necessity for new biomarkers that help to improve the survival prediction and tailor therapies more rationally and precisely. In the present study, we established a stroma‐related lncRNA signature (SLS) based on 52 lncRNAs to comprehensively predict clinical outcome. The SLS model could not only distinguish patients with different recurrence and mortality risks through univariate analysis, but also served as an independent factor for relapse‐free and overall survival. Compared with the conventionally used TNM stage system, the SLS model clearly possessed higher predictive accuracy. Moreover, the SLS model also effectively screened chemotherapy‐responsive patients, as only patients in the low‐SLS group could benefit from adjuvant chemotherapy. The following cell infiltration and competing endogenous RNA (ceRNA) network functional analyses further confirmed the association between the SLS model and stromal activation‐related biological processes. Additionally, this study also identified three phenotypically distinct colon cancer subtypes that varied in clinical outcome and chemotherapy benefits. In conclusion, our SLS model may be a significant determinant of survival and chemotherapeutic decision‐making in colon cancer and may have a strong clinical transformation value.

## 1 INTRODUCTION

Colon cancer is one of the commonest cancers in the world. The surgical adjuvant chemotherapy (ADJC) based on combination of oxaliplatin, fluorouracil and leucovorin is an effective option in clinical practice of patients with stage II‐III colon cancer,^1^, ^2^ and over 80% of relapse cases developed within 3 years of the initial primary resection.^3^ Currently, the American Joint Committee on Cancer TNM staging system, which assesses tumour invasion depth, lymph node metastatic status and remote metastasis,^4^ is still the most commonly used indicator for assessing the recurrence risk of patients with colon cancer and whether they need ADJC or not. However, the predictive ability of this system is considered insufficient, as it does not predict the outcome of the patients precisely enough. Owing to high levels of heterogeneity found in colon cancer, prognoses and chemotherapeutic responses may vary widely between patients with similar clinical features. Therefore, it is necessary to develop novel biomarkers to help clinical workers tailor therapies more rationally and precisely.

Accumulating evidence has suggested that genetic difference of tumours is the major cause of heterogeneous anti‐cancer drug response.^5^, ^6^ However, previous efforts to develop models for predicting chemotherapy response based on expression and mutation profiling of protein‐coding genes (PCGs) have been unsuccessful.^7^, ^8^ Similar to PCGs, long non‐coding RNAs (lncRNAs), which used to be regarded as ‘transcript junk’,^9^, ^10^ also act as key regulators that participate in multiple biological processes involved in tumour development, progression and cancer therapy response.^6^, ^11^ LncRNA is a transcript longer than 200 nucleotides that cannot be translated into proteins^10^ and is among the most prevalent transcriptional changes in tumour.^11^ In colon cancer, several prognostic predictive models have been developed based on lncRNAs.^12^, ^13^, ^14^, ^15^ However, as none of them has been reported to provide potential treatment guidance, these models may not meet clinical needs. Nevertheless, the clinical significance of lncRNAs in colon cancer still needs further exploration.

Here, we downloaded five datasets of colon cancer derived from Gene Expression Omnibus (GEO; http://www.ncbi.nlm.nih.gov/geo/), which consisted of the transcriptome profile data of 988 samples. To establish a lncRNAs‐based model that could be used for improving the relapse risk prediction and tailoring therapies, we specially identified lncRNAs that were significantly associated with both cancer prognosis and biological processes including angiogenesis, hypoxia, TGFβ signalling and epithelial‐mesenchymal transformation (EMT), which have been well‐studied in multiple solid tumours, and defined as important stroma‐related factors mediating tumour metastasis and drug resistance, including in colon cancer.^16^, ^17^, ^18^

## 2 METHODS

### 2.1 Data source, study population and clinicopathological variables

The workflow chart is shown in Figure S1. A summary of all dataset information used in this study is provided in Table S1. Sample tissues were excluded from the study if they came from a normal colon, stage IV patients, or patients without survival information on relapse. Then, all eligible samples were randomly separated into the training and validation (7:3) set groups using the ‘caret’ package. In addition, for gastric cancer exploration, the ‘http://www.ncbi.nlm.nih.gov/geo/query/acc.cgi?acc=GSE62254’ dataset was also downloaded and analysed. The demographic information and clinical information were retrieved using the ‘GEOquery’ package for GEO datasets. The end‐points analysed in this study were relapse‐free survival (RFS), defined as the interval between the date of diagnosis and date of tumour relapse, and overall survival (OS), defined as the interval between the date of diagnosis and death.

### 2.2 Microarray data processing and lncRNA profile mining

As all samples involved in the downloaded colon cancer datasets were hybridized to Affymetrix HG‐U133 Plus 2.0, the raw microarray data were all renormalized using a robust multiarray averaging method with ‘affy’ and ‘simpleaffy’ packages. The ‘ComBat’ algorithm was applied to reduce the likelihood of batch effects from non‐biological technical biases. The lncRNA annotations were collected from three sources: (a) Based on the ‘Comprehensive gene annotation’ file provided by the GENCODE website, we screened the official gene annotation documents of the Affymetrix HG‐U133 Plus 2.0 platform for the lncRNA gene; (b) all GPL570 platform probes were mapped to the ‘Transcript sequences’ file downloaded from the GENCODE website using SeqMap and re‐annotated with ensembl ID.^19^ Then, the lncRNA probes were extracted based on a gene transfer format file for ‘Long non‐coding RNA gene annotation’. Of note, the probes were dropped if the lncRNA gene type was marked as a TEC gene; and (c) lncRNA annotation files generated by Zhang et al were also downloaded and checked as a supplement.^20^ Finally, 4037 distinct annotated lncRNA transcripts with corresponding Affymetrix probe IDs were generated.

### 2.3 Determination of lncRNA function

lncRNA function was explored using the triple competing endogenous RNA (lncRNA‐miRNA‐mRNA) network^21^ constructed through the following steps: First, we predicted the miRNA target of each lncRNA using the miRCODE website; then, the corresponding PCGs were identified using miRDB, miRTarBase and TargetScan; the correlation between lncRNA and PCGs was further tested by calculating the Pearson correlation coefficients. The PCGs significantly positively correlated with lncRNA (correlation coefficient > .5, adjusted *P* value < .001) were considered lncRNA‐related PCGs, and these genes were entered into the Gene Ontology (GO) enrichment analysis to determine the lncRNA function. Finally, we chose 50 lncRNA‐related PCGs with the highest correlation as representatives to draw the triple ceRNA network using Cytoscape software, to show the interaction between genes more clearly.

### 2.4 Gene set variation analysis (GSVA)

Gene set variation analysis is the most often used method to estimate biological process activity.^22^ In the present study, gene sets for ‘EMT’, ‘angiogenesis’, ‘hypoxia’ and ‘TGFβ signalling’ were retrieved from the ‘hallmark gene sets’ collection of the ‘molecular signatures database’ and were employed for GSVA using the ‘GSVA’ package. The sum of the GSVA score obtained from the above four gene sets was defined as the stromal activation level of the corresponding sample.

### 2.5 Characterization of tumour microenvironment

To quantify the infiltration levels of stromal cells located in tumour microenvironment, the single‐sample gene set enrichment analysis (ssGSEA) method was applied using the ‘GSVA’ package.^23^ The marker genes of immune and stromal cells used for ssGSEA were retrieved from the works of Bindea et al^24^ (24 types of innate and adaptive immune cells covering multiple functional subtypes, and blood and lymph vessel cells) and Becht et al^25^ (endothelial cells and fibroblasts). The stromal score developed by Yoshihara et al was calculated using the ‘ESTIMATE’ R package.^26^

### 2.6 LASSO Cox regression and survival analysis

The least absolute shrinkage and selection operator (LASSO) regression is a commonly used method for feature selection and has been applied to the Cox proportional hazard regression model.^27^ As previously described,^28^, ^29^ only lncRNAs which passed the 1000‐times bootstrapping robustness test were selected for LASSO regression analysis and all lncRNA expression values were dichotomized and respectively assigned as 1(represents lower expression) and 2 (represents higher expression). The genes represented by the minimum penalty parameter, λ, would be chosen to establish the prognostic risk score formula via Cox regression analysis in the training cohort. The optimal cut‐off values for each gene were calculated using the ‘survminer’ R package. The Kaplan‐Meier method was applied to calculate the survival rate, and the log‐rank test was performed to assess the statistical significance. Uni‐ and multivariate analyses were performed using the Cox proportional hazard models with a stepwise ‘LR forward’ method. The time‐dependent receiver operating characteristic curve (TDROC) and Harrell's concordance index (c‐index) analyses were conducted to evaluate the predictive value of the prognostic models.

### 2.7 Other statistical analyses

The ‘CancerSubtypes’ R package was used for identifying molecular cancer subtypes based on aggregating multiple genomic platform data.^30^ The chemotherapy response of each sample was predicted by a predefined FOLFIRI (a chemotherapy regimen combination of irinotecan, 5‐fluorouracil and leucovorin deployed in first‐line treatment of patients with metastatic colon cancer) response signature^31^ using the nearest template prediction (NTP) algorithm^32^ module from GenePattern as described by Sadanandam et al.^33^ The unpaired Student's *t* test (two groups) and one‐way ANOVA test (more than two groups) were used for normally distributed data comparison; otherwise, the Mann‐Whitney U test (two groups) and Kruskal‐Wallis test (more than two groups) were performed. Nomogram construction and validation were performed following Iasonos’ guide.^34^ All statistical analyses were conducted using R software (version 3.5.0) and SPSS software (version 25.0), and *P* values were two‐tailed. Statistical significance was set at *P* < .05.

## 3 RESULTS

### 3.1 Identification of robust prognostic stroma‐related lncRNAs

The patient cohort of this research included 988 patients. Pearson's correlation test identified a total of 2654 lncRNAs significantly related with the stromal activation level (adjusted *P* value < .05). Among them, 372 lncRNAs were significantly correlated with RFS via the Cox univariate regression analysis in the entire cohort. Next, we used 1000‐times bootstrapping to test the predictive robustness of the above 372 genes as we mentioned in ‘Methods’ section. Finally, 82 lncRNAs, the expression levels of which were stably and significantly correlated with prognosis, were identified and defined as robust prognostic stroma‐related lncRNAs (see Table S2).

### 3.2 The stroma‐related lncRNA signature (SLS) generation and validation

**Figure 1:**
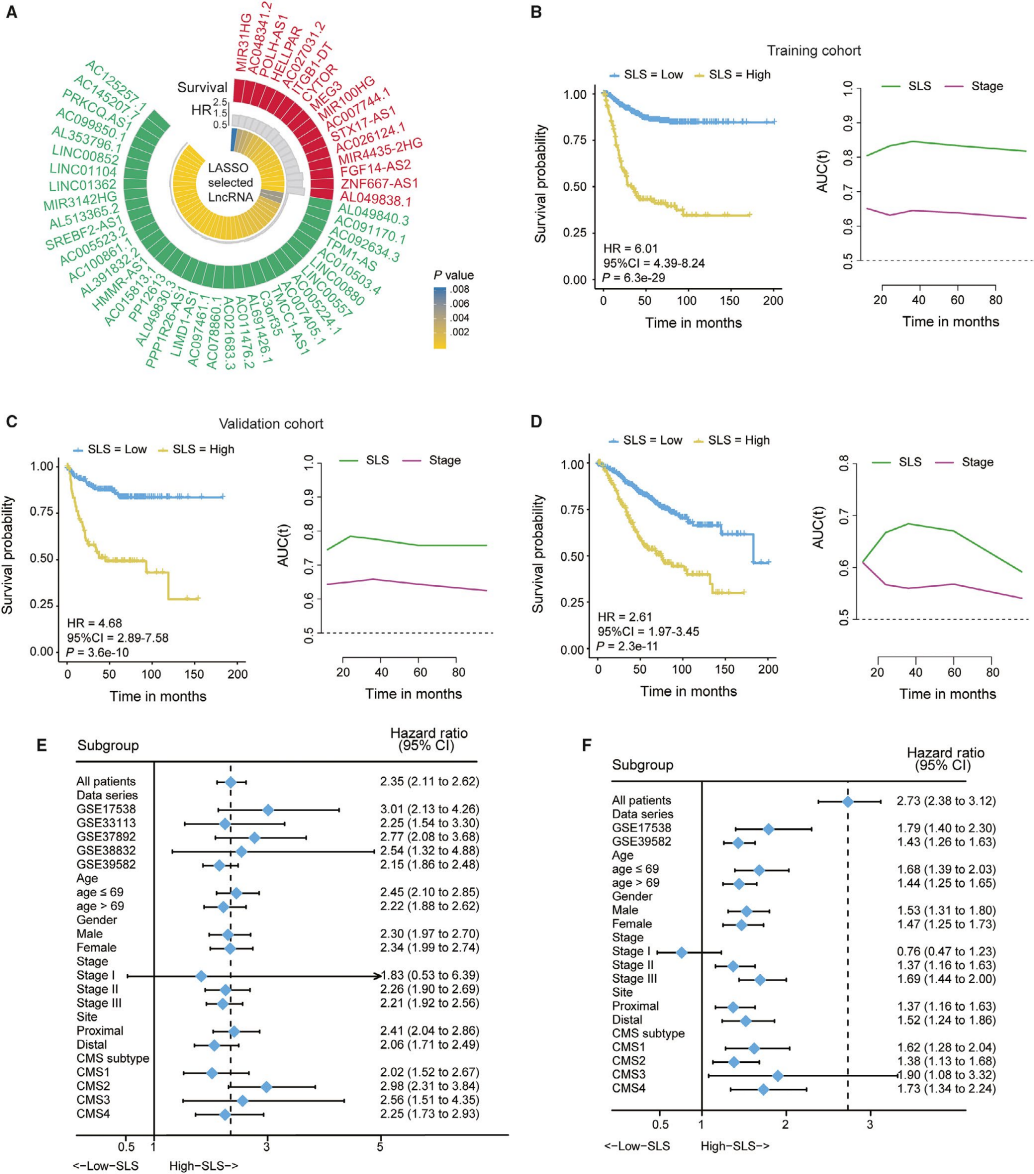
The survival impact of SLS model. A, Circos plot shows the survival impact of 52 lncRNAs selected by LASSO Cox regression analysis (risk factor, red; protect factor, green); B‐C, Kaplan‐Meier curves (left) and TDROC curves (right) of relapse‐free survival according to SLS groups in the training cohort; (B) and validation cohort (C); D, Kaplan‐Meier curves (left) and TDROC curves (right) of overall survival according to SLS groups; E‐F, Forest plots of the associations between SLS and relapse‐free survival (E) and the associations between SLS and overall survival (F) in various subgroups. Unadjusted HRs (boxes) and 95% confidence intervals (horizontal lines) are depicted. AUC, area under ROC curve; CI, confidence interval; CMS, consensus molecular subtypes; HR, hazard ratio; SLS, stroma‐related lncRNA signature

The 988 colon cancer samples were randomly regrouped into training (n = 692) and validation cohorts (n = 296) as described in the ‘Methods’ section. Comparison of patient characteristics between the two groups showed no significant differences (*P* > .05; see Table S3). Through LASSO analysis (see Figure S2), 52 lncRNAs were screened out as predictors to generate the SLS model and their survival impact is shown in Figure 1A. The cut‐off values and risk coefficients of these 52 lncRNAs used for creating the risk score formula are listed in Table S4. The patients were divided into two groups based on the optimal cut‐off values of the SLS model obtained for the entire cohort (−8.49). In both training and validation sets, patients in the high‐SLS group had a significantly shorter RFS time (Figure 1B‐C) and the predictive ability for relapse of the SLS model was obviously higher than that of the TNM stage system, revealed by both TDROC (Figure 1B‐C) and c‐index analyses (Table 1). The association of the SLS risk score with RFS was also tested in the multivariate Cox regression model as a continuous variable. As shown in Table 2, the SLS model was a demonstrably strong independent risk factor for RFS in both patient cohorts. Similar results were also found for the analysis of 678 patients with documented OS information (Figure 1D, Tables 1 and 2). In addition, we also conducted subgroup analyses to further validate the prognostic role of the SLS model. The forest plots indicated that regardless of whether it was referring to RFS (Figure 1E) or OS (Figure 1F), a higher SLS score was significantly associated with a poorer prognosis in all subgroups but the stage I patients.

**Table 1:** Harrell's concordance indexes of the SLS model and stage in different cohorts

Survival	Cohort	SLS^a^	Stage^6th^
RFS	Training	0.80 ± 0.03	0.62 ± 0.04
	Validation	0.74 ± 0.06	0.63 ± 0.06
	Entire	0.79 ± 0.03	0.63 ± 0.03
OS	Entire	0.65 ± 0.04	0.55 ± 0.04

Abbreviation: OS, overall survival; RFS, relapse‐free survival; SLS, stroma‐related LncRNA signature.

a Continuous variables.

**Table 2:** Univariate and multivariate survival analyses of SLS model and clinical variables

	UVA (RFS)		UVA (OS)		MVA (RFS)				MVA (OS)	
	Entire	*P* value	Entire	*P* value	Training	*P* value	Validation	*P* value	Entire	*P* value
Age^a^	1.00 (0.99‐1.01)	.996	1.04 (1.02‐1.05)	<.001	NE		NE		1.03 (1.02‐1.05)	<.001
Gender (vs Male)	0.73 (0.59‐0.90)	.003	0.82 (0.62‐1.09)	.176					NE	
SLS^a^	2.35 (2.11‐2.62)	<.001	1.50 (1.34‐1.68)	<.001	2.58 (2.16‐3.09)	<.001	1.99 (1.58‐2.52)	<.001	1.58 (1.40‐1.78)	<.001
Stage (vs stage I)										
Stage II	8.24 (2.03‐33.39)	.003	1.74 (0.91‐3.34)	.097	5.36 (1.30‐22.09)	.020	NE		NE	
Stage III	17.97 (4.45‐72.55)	<.001	2.30 (1.20‐4.41)	.012	7.66 (1.87‐31.37)	.005				
CMS (vs CMS4)										
CMS1	0.55 (0.35‐0.86)	.009	0.96 (0.63‐1.45)	.846	NE		NE		NE	
CMS2	0.59 (0.41‐0.84)	.003	0.63 (0.44‐0.92)	.015						
CMS3	0.42 (0.25‐0.73)	.002	0.38 (0.20‐0.72)	.003						

Abbreviation: CMS, consensus molecular subtypes; MVA, multivariate analysis; NE, not enter; OS, overall survival; RFS, relapse‐free survival; SLS, stroma‐related lncRNA signature; UVA, univariate analysis.

a Continuous variable.

### 3.3 SLS and the therapeutic benefit of adjuvant chemotherapy

**Figure 2:**
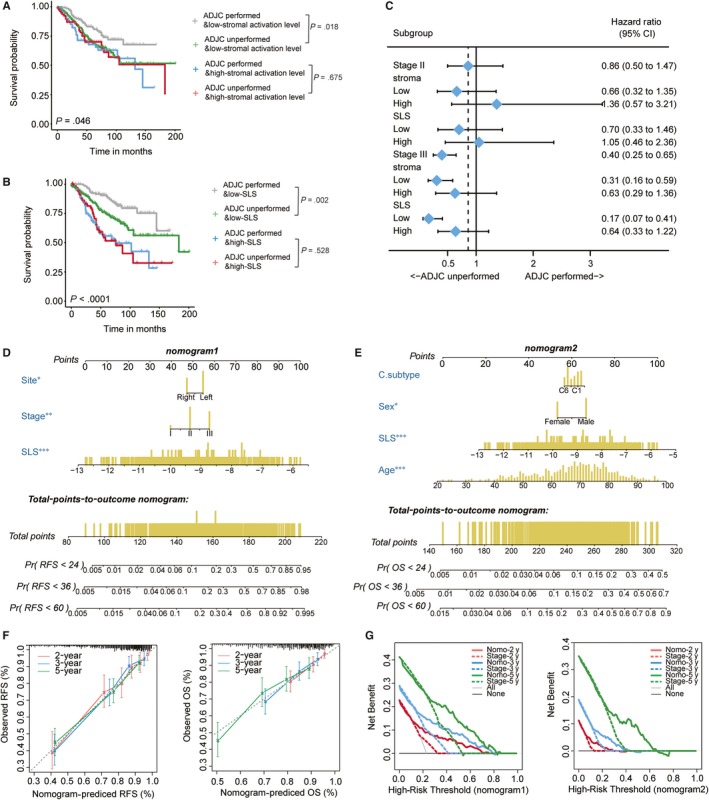
The predictive effect of SLS model on treatment outcome of adjuvant chemotherapy. A‐B, Kaplan‐Meier curves of overall survival for patients in subgroups stratified by both stromal activation level (A)/SLS model (B), and receipt of adjuvant chemotherapy; C, Forest plot showing the benefit of adjuvant chemotherapy in different subgroups stratified by both stromal activation level/SLS model, and stage; D‐E, Nomograms incorporating SLS model and clinical variables for predicting patient relapse (D) and death (E); F, Calibration plots show the agreement of prediction performance of nomogram and ideal model (45‐degree dotted line); G, Decision curve plots depict the clinical usefulness of the nomograms and TNM stage. ADJC, adjuvant chemotherapy; OS, overall survival; Pr, probability; RFS, relapse‐free survival; SLS, stroma‐related lncRNA signature

Several studies have reported that EMT, TGFβ signalling, angiogenesis and hypoxia are all important biological processes influencing chemotherapy efficacy. Correspondingly, through analysing the http://www.ncbi.nlm.nih.gov/geo/query/acc.cgi?acc=GSE39582 dataset, we found that patients with a high stromal activation level could not benefit from adjuvant chemotherapy (ADJC) (Figure 2A). As the SLS model consisted of stromal activation level‐related lncRNAs, we were interested in knowing whether the SLS model also helps to find patients who could potentially benefit from chemotherapy. As expected, ADJC could only significantly reduce the mortality risk of patients in the low‐SLS group, but did not confer survival benefits to patients in the high‐SLS group (Figure 2B). Moreover, the survival benefit of ADJC was more obvious for patients with a low‐SLS value in both stage II and stage III subgroups, although the *P* value did not reach a statistical significance in stage II patients (Figure 2C). We then conducted multivariate analyses in the http://www.ncbi.nlm.nih.gov/geo/query/acc.cgi?acc=GSE39582 dataset (see Table S5) and obtained the independent prognostic factors for RFS and OS, respectively, to construct the nomograms (Figure 2D‐E). The calibration plots showed that both nomograms performed well compared with the performance of the ideal models (Figure 2F). Decision curve analysis revealed that the clinical usefulness of the nomograms significantly overwhelmed that of the TNM stage system (Figure 2G).

### 3.4 The relationship between SLS signature, tumour microenvironment, clinical parameters and biological processes

**Figure 3:**
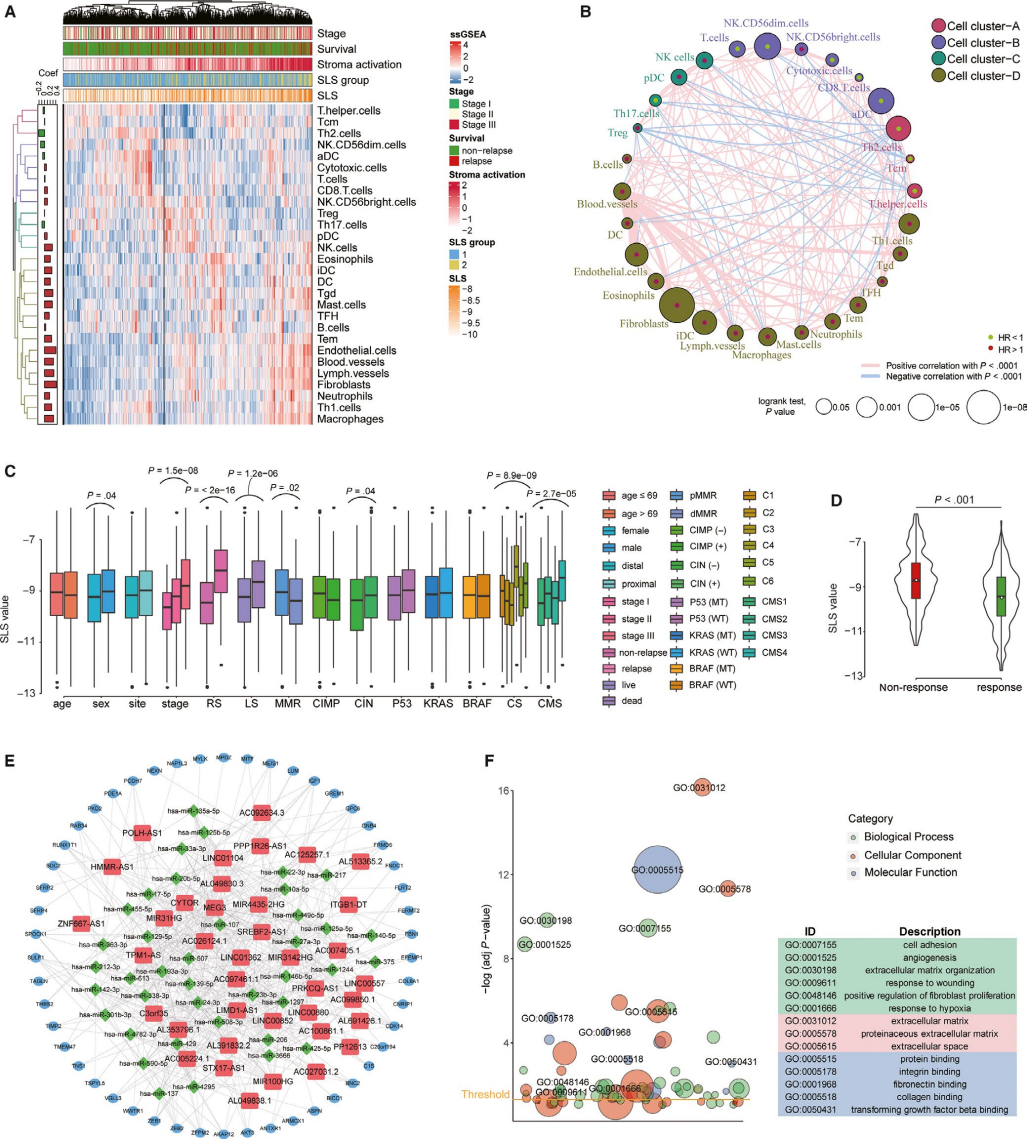
Cell infiltration, clinical significance and biological function analyses of SLS model. A, Unsupervised clustering of patients based on 28 types of tumour microenvironment cell infiltration which characterized by single‐sample gene set enrichment analysis. Patient stage, relapse status, stroma activation level, SLS group and SLS value were annotated above the heat map. Four distinct cell clusters are defined; B, The interactions between each cell and their survival impact. The size of each cell represents survival impact of each cell type, calculated using the formula log10 (log‐rank test *P* value). The lines connecting cells represent cellular interactions. The thickness of line represents the strength of correlation estimated by Spearman's correlation analysis. Favour for relapse‐free survival was coloured as green, risk for relapse‐free survival as red; C‐D, SLS values in different clinical subgroups (C) and in patient group with different response to FORFIRI (D); E, The overall ceRNA network of lncRNAs. In the network, protein‐coding genes are coloured in blue, miRNAs are coloured in green and lncRNAs are coloured in red; F, The bubble plot shows the GO enrichment results of lncRNA‐related protein‐coding genes.aDC, activated dendritic cell; CIMP, CpG island methylator phenotype; CIN, chromosome instability; CMS, consensus molecular subtypes; CS, c subtype; DC, dendritic cell; iDC, immature dendritic cell; LS, live status; MMR, mismatch repair; MT, mutant type; RS, relapse status; SLS, stroma‐related lncRNA signature; T gamma delta; Tcm, T central memory; Tem, T effector memory; TFH, T follicular helper; Tgd, NK, natural killer; Th, T helper; WT, wild type

The correlation between SLS signature and tumour microenvironment (TME) landscape was evaluated. Using unsupervised clustering, we identified four distinct TME infiltrated cell clusters (Figure 3A), and the survival impact of each cells is exhibited in Figure 3B. As shown in the heat map, the cells whose infiltration levels were significantly positively associated with SLS scores mainly fell into cell cluster D, which was characterized by a high infiltration level of multiple stromal cells, macrophages, neutrophils, eosinophils and some T‐cell subtypes (fibroblasts and endothelial cells were of the highest correlation level). An increased infiltration level of these cells indicated a poor prognosis. Contrarily, cells that were significantly negatively associated with SLS mainly included activated dendritic cell (aDC), CD56^dim^ natural killer (NK) cells, Th17 cells and Th2 cells, which acted as protective prognostic factors. Exploring the clinical indications of SLS showed that male, advanced stages, relapse or death, proficient mismatch repair status and chromosome instability‐positive patient characteristics all had significantly raised SLS scores (Figure 3C). In terms of molecular subtypes, patients in molecular subtypes C4 and CMS4 exhibited significantly higher SLS model values than others. Moreover, in consistency with the results of ADJC analysis, we also found that the SLS score was significantly higher in patients that were classified into a FOLFIRI‐insensitive group compared with a FOLFIRI‐sensitive group (Figure 3D). Finally, we built the triple ceRNA (lncRNA‐miRNA‐mRNA) network (Figure 3E) to further determine the function of lncRNAs involved in SLS models. The following GO functional analysis of the PCGs in the ceRNA networs also showed the enrichment of multiple stromal activation‐related terms (Figure 3F), including cell adhesion to various extracellular matrix component, angiogenesis and hypoxia.

### 3.5 Evaluation of novel colon cancer subtypes by aggregating lncRNA and mRNA data

**Figure 4:**
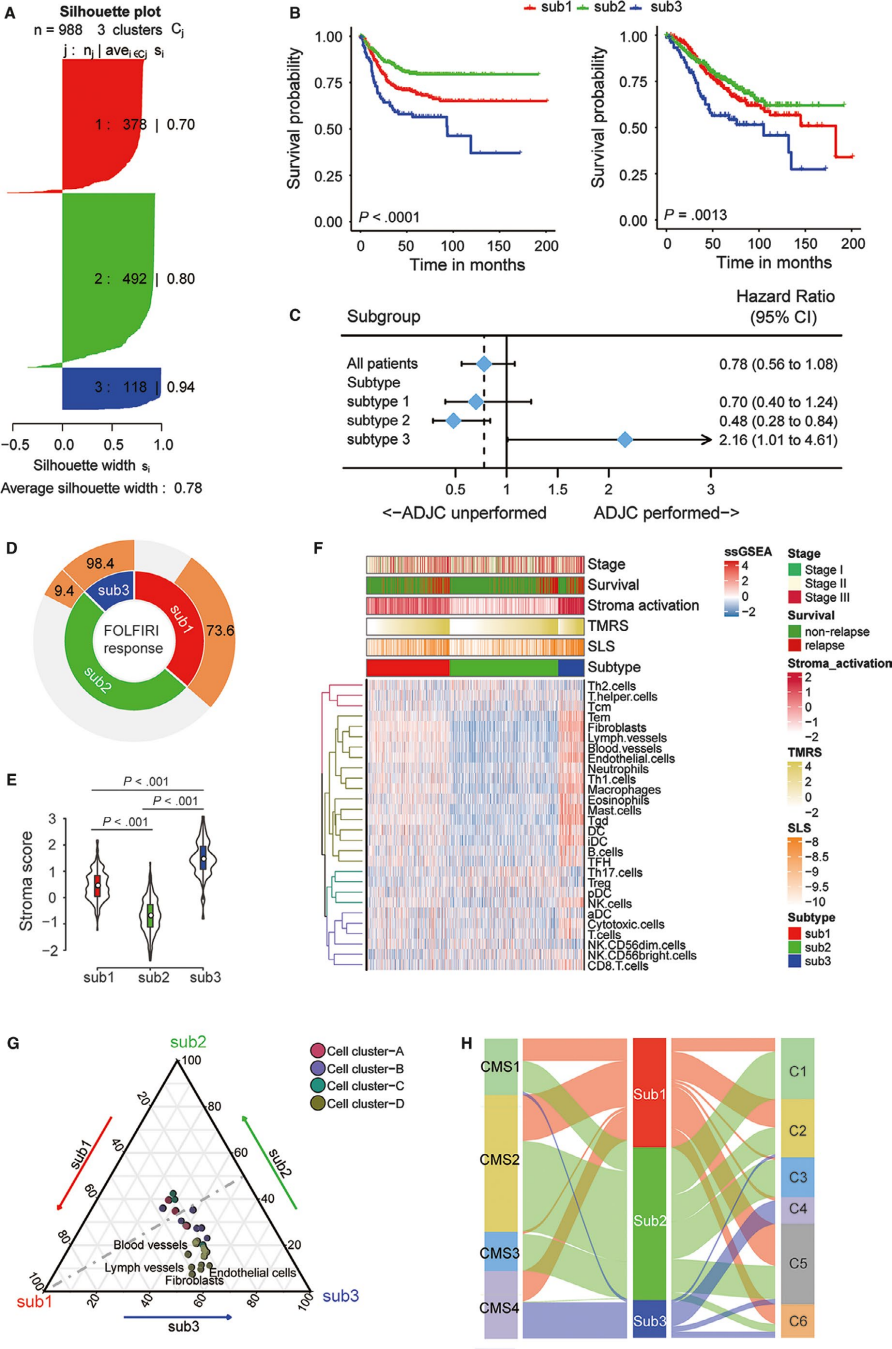
Unsupervised clustering of lncRNAs (from SLS) and mRNAs (from TMRS) in colon cancer. A, Average silhouette width representing the coherence of clusters; B, The survival curve of three distinct subtypes for relapse‐free survival (left) and overall survival (right); C, Forest plot showing the benefit of adjuvant chemotherapy in different subtypes; D, Sunburst chart depicting the non‐response rate to FOLFIRI in different subtypes; E, Stroma score in different subtypes; F, Heat map shows the tumour microenvironment cell infiltration status in different subtypes. Patient stage, relapse status, stroma activation level, TMRS value, SLS value and cancer subgroup were annotated above the heat map. Four distinct cell clusters are defined as previous; G, Ternary plot of infiltration status in cell clusters comparing different subtypes; H, Sankey chart displaying the distribution of the novel subtypes in C1‐C6 subtypes and CMS subtypes. aDC, activated dendritic cell; CMS, consensus molecular subtypes; DC, dendritic cell; iDC, immature dendritic cell; Sub, subtype; T gamma delta; Tcm, T central memory; Tem, T effector memory; TFH, T follicular helper; Tgd, NK, natural killer; Th, T helper

In a previous study, we identified 100 robust prognostic TME genes and found that a panel consisting of these genes could not only precisely predict the relapse and mortality risks in patients with colon cancer, but also discriminate patients who would potentially benefit from ADJC.^28^ However, it has been widely believed that aggregating multiple types of genomic data could help to reflect the complexity of the tumour genetic characteristics. Therefore, we attempted to construct a novel molecular subtype based on both the 52 stroma‐related lncRNAs identified in this study and 100 TME genes. We chose three as the optimal clustering number (see Figure S3) and samples were then divided into three distinct subtypes based on SNF‐CC approach (combination of ‘similarity network fusion’ method and ‘consensus clustering’ method) 30 using ‘CancerSubtypes’ R package (Figure 4A). The survival analyses revealed a significant survival difference between the three subtypes: The relapse and death risks were highest in subtype 3, whereas subtype 2 showed the best prognosis (Figure 4B). The chemotherapy treatment effect analysis (Figure 4C) showed that only patients from subtype 2 could significantly benefit from ADJC, whereas subtype 1 and subtype 3 patients could not. In particular, for subtype 3 patients, the ADJC conduction was a risk factor for an unpromising prognosis. We then, respectively, compared the FOLFIRI response rate (Figure 4D) and stroma score (calculated by ESTIMATE algorithm, Figure 4E) between the different patient subtypes. The result showed that both the non‐response rate and the stroma score were also highest in subtype 3 patients and lowest in subtype 2 patients. Referring to cell infiltration, both the heat map (Figure 4F) and triangle plot (Figure 4G) showed that it was cell cluster D, including multiple stromal cells, that exhibited the significant difference of infiltration between the three subtypes. Finally, we evaluated the relationship between the current classification with other established colon cancer molecular subtypes (CMS subtype and C subtype). As shown by the Sankey plot (Figure 4H), the subtype 3 patients mainly fell into the CMS4 subtypes, mostly representing mesenchymal phenotypes, whereas the subtype 2 patients mainly fell in CMS2, mostly displaying epithelial phenotype characteristics. Similarly, the samples in subtype 2 were distributed in the C subtypes apart from C4 and C6 (both characterized by EMT pathway upregulation) subtypes, whereas subtype 3 samples were mainly distributed in C4.

### 3.6 The role of SLS in gastric cancer

**Figure 5:**
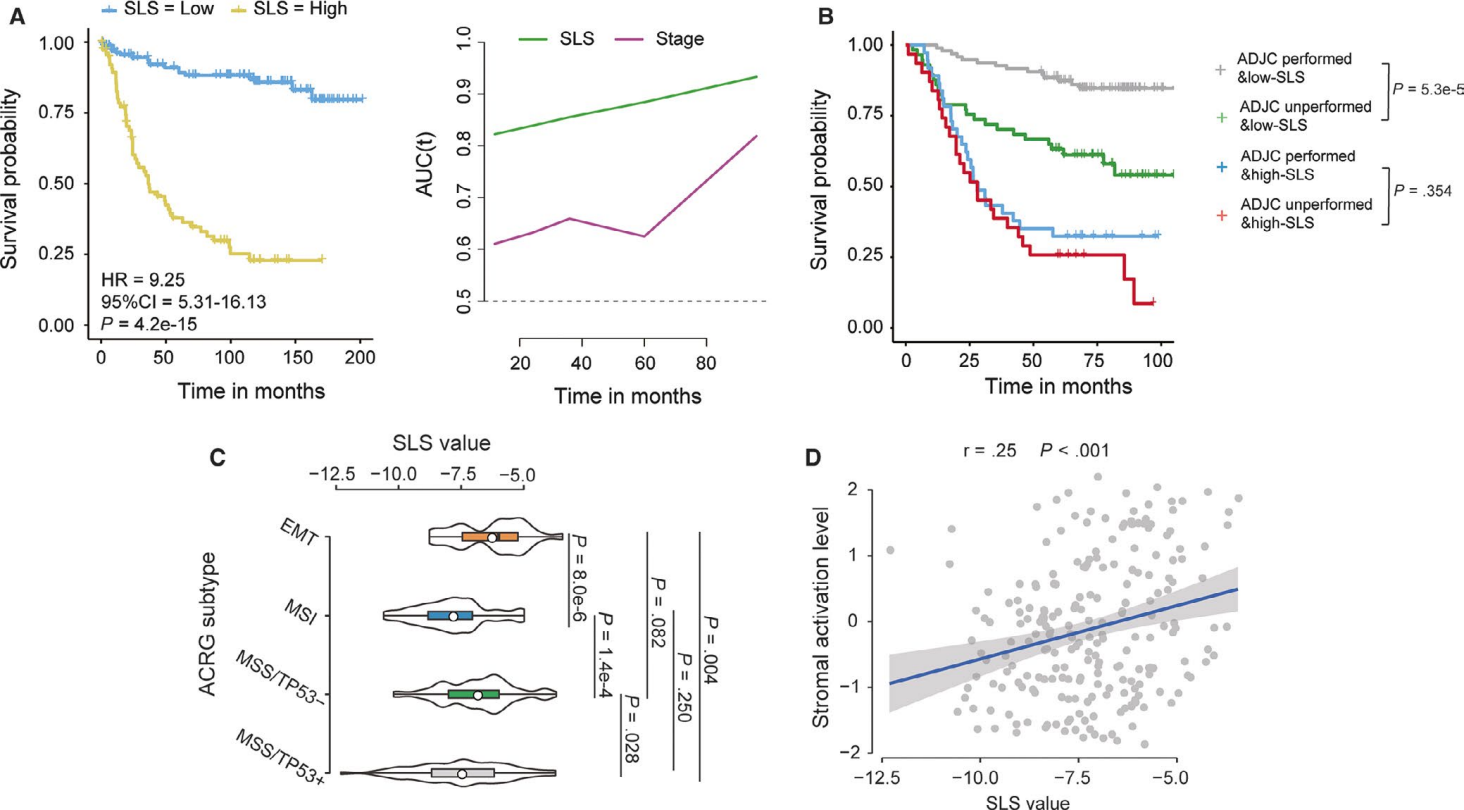
The role of SLS model in gastric cancer. A, Kaplan‐Meier curves (left) and TDROC curves (right) of relapse‐free survival according to SLS groups; B, Kaplan‐Meier curves of overall survival for patients in subgroups stratified by both SLS model and receipt of adjuvant chemotherapy; C, SLS values in different ACRG subtypes; D, Scatter plot showing the correlation between SLS value and stomal activation level. ADJC, adjuvant chemotherapy; AUC, area under ROC curve; SLS, stroma‐related lncRNA signature

We also analysed the role of the SLS model in the http://www.ncbi.nlm.nih.gov/geo/query/acc.cgi?acc=GSE62254 database to verify whether it was also correlated with relapse and ADJC treatment effect in patients with gastric cancer. The clinical characteristics of this cohort were also listed (see Table S6). The cut‐off values for each lncRNA were re‐calculated because of the potential existence of heterogeneity between different tumour types. As shown in Figure 5A‐B, this dataset confirmed the ability of the SLS model in predicting survival and tailoring therapies for patients with stage I‐III gastric cancer. In the multivariable Cox regression model, the SLS model also was significantly associated with RFS as a continuous variable in the http://www.ncbi.nlm.nih.gov/geo/query/acc.cgi?acc=GSE62254 cohort (see Table S7). Finally, we compared the SLS scores of gastric cancer patients with different Asian Cancer Research Group subtypes. The results showed that the SLS scores were significantly higher in the ‘EMT’ and ‘MSS/TP53‐’ subtypes compared with the ‘MSI’ and ‘MSS/TP53+’ subtypes (Figure 5C). The following scatter plot also confirmed that SLS scores were significantly positively associated with stromal activation level in patients with gastric cancer (Figure 5D).

## 4 DISCUSSION

The rapid development of high‐throughput technology has enabled researchers to study cancer heterogeneity more deeply and comprehensively, and identify ideal molecular biomarkers that could be used to precisely predict prognosis and guide treatment. Among them, lncRNAs, which possess tissue‐specific and cancer‐specific expression patterns and play essential roles in tumorigenesis and cancer progression,^11^ are specifically regarded as important candidates for building diagnostic and prognostic signatures in various types of solid tumours. In colon cancer, several lncRNA‐based prognostic signatures have been established to improve survival prediction. For example, the 4‐lncRNA signature proposed by Wang et al,^14^ the 2‐lncRNA signature built by Xue et al^15^ and the 15‐lncRNA signature developed by Wang et al^13^ were all reported to be an ideal tool to identify colon cancer patients with a high mortality risk. Most recently, Cai's group developed an integrated mRNA‐lncRNA signature with predictive values specially for early colon cancer relapse (recurrence between 3 months and 1 year after initial primary resection) and found that the prognostic ability of the model was stronger than that of the TNM stage system.^12^ However, the current available signatures only provide prognosis‐related information; none of these studies discussed the role of these signatures in treatment guidance. The clinical practicality of these signatures was therefore limited.

To construct an lncRNA‐based model that could provide information on patient prognosis and therapeutic benefit, we first used correlation analysis to obtain 2654 lncRNAs that were significantly associated with the biological processes that trigger tumour metastasis and chemotherapy resistance, including angiogenesis, hypoxia, EMT and TGFβ signalling activation.^16^, ^17^, ^18^ Then, by consistently using the bootstrap and LASSO regression methods, we selected 52 robust stroma‐related prognostic lncRNAs to build a novel prognostic model, the SLS model. The following survival and prediction power analyses revealed that the SLS model was a reliable tool for prognostic prediction and had much better predictive accuracy than the AJCC TNM stage system. More importantly, by applying the SLS signature to the http://www.ncbi.nlm.nih.gov/geo/query/acc.cgi?acc=GSE39582 dataset, we noticed that only patients in a low‐SLS group could benefit from ADJC, which indicated that the SLS model could effectively screen patients who are chemotherapy‐responsive. We also uncovered a difference in SLS value between FOLFIRI‐sensitive and FOLFIRI‐insensitive groups, which further implied that our SLS model had the potential to differentiate between chemotherapy benefits in either adjuvant or metastatic settings. These findings suggested that the primary chemotherapy resistance caused by high activation levels of stroma‐related biological process could be one reason for the unpromising prognosis of high‐SLS patient; the development of new therapeutic models by a combination of chemotherapy and molecule‐targeted drugs that could inhibit activation of specific stroma‐related pathways might be helpful for improving their curative effect. Moreover, the SLS signature also showed excellent performance in recurrence prediction and the identification patients for whom ADJC may be more beneficial in gastric cancer, indicating a broad applicability of this signature in gastrointestinal cancer. Taken together, our results suggest that the SLS signature may be a significant determinant of survival and chemotherapeutic decision‐making in colon cancer and have a strong clinical transformation value. Of note, due to limitations of the sample size in the datasets, additional prospective studies are required to further verify our findings.

In this study, we also constructed a new colon cancer molecular subtype by SNF‐CC method that aggregates the expression data of 52 lncRNAs and 100 robust prognostic TME genes identified in our previous study.^28^ Similar to SLS signature, this derived subtype was also associated with a significant difference in survival outcome and the curative effect of ADJC and FOLFIRI regimens, indicating that such a genomic classification offers insights to the stratified management of patients and further personalized therapeutic decision‐making. Notably, in subtype 3 patients, ADJC did not benefit patients and even increased the mortality risk by 2.16 times. We speculated that it might be due to the fact that chemotherapy itself increased the metastatic potential of the drug‐resistant cells, and accelerated tumour progression.^35^, ^36^ Therefore, research on developing new strategies for subtype 3 patients is warranted.

Among the 52 lncRNAs involved in the SLS signature, 14 have been experimentally demonstrated to be linked with cancer. Therein, four lncRNAs including CYTOR, MEG3, MIR100‐HG and MIR31HG have been previously reported to play an oncogenic role in colon cancer. Wang et al reported that CYTOR could directly interact with NCL and Sam68, and the latter two further activated the NF‐κB pathway and EMT to contribute to colon cancer progression.^37^, ^38^ MEG3 level has been widely reported to decrease in a growing list of primary human tumours and plays a key role in tumour suppression. In colon cancer, down‐regulation of lncRNA MEG3 promotes cancer cell proliferation and migration via upregulating clusterin,^39^TGF‐β1 and SPHK1.^40^ MIR100HG is a kind of microRNA host gene, the intron of which encodes three kinds of microRNA, including miR‐100, miR‐125b‐1 and let‐7a‐2. There has only been one study showing that MIR100HG could form a double‐negative feedback loop with GATA6 and activate the Wnt/β‐catenin pathway, causing cetuximab resistance in colon cancer cells.^41^ As for MIR31HG, Eide et al found that MIR31HG was an independent prognostic factor for patients with colon cancer.^42^ Meanwhile, the cell lines with high MIR31HG outlier expression were characterized by an elevated EMT signature and TGFβ signalling activation.^42^ This result is consistent with our findings. However, the functions of most lncRNAs enrolled in the signature have not been investigated. As our research has shown that the expression of these lncRNAs was correlated with stroma activation level and robustly associated with increased recurrence risk, they deserve further clinical and basic investigation.

Limitations to our study include the following: First, as this study was conducted based on publicly available datasets, some important clinicopathological features were not available for analysis and could lead to potential error or bias. Second, all colon cancer transcriptome profiling collected in the present study was produced by the GPL570 platform; the lncRNA candidates identified here may not represent the complete lncRNA populations underlying colon cancer biological behaviour, owing to the fact that the GPL570 platform only contains a small part of all possible lncRNAs. Moreover, the lncRNAs that were identified by re‐annotation of probes should also be validated by further studies. Finally, as mentioned above, the biological function of some lncRNAs involved in SLS signatures still requires experimental verification.

## 5 CONCLUSIONS

In conclusion, our study developed a novel lncRNA signature that can be used as a reliable tool for personalized prognosis prediction and for treatment decision‐making in patients with colon cancer. Further prospective clinical trials are warranted to validate our findings.

## CONFLICT OF INTEREST

The authors declare that they have no competing interests.

## AUTHORS' CONTRIBUTIONS

RZ, HS and WL contributed to the planning of the study and drafted the manuscript. ZS participated in manuscript revision and verified the numerical results by an independent implementation. RZ, HS, JZ, DZ and JW prepared all the figures and tables. JW, ZH, XR, JB, YL and MS contributed to interpretation of data and reviewing of the manuscript. All the authors reviewed and approved the final manuscript.

## Data Availability

Availability of supporting data: http://www.ncbi.nlm.nih.gov/geo/query/acc.cgi?acc=GSE17536 (http://www.ncbi.nlm.nih.gov/geo/query/acc.cgi?acc=GSE17536); http://www.ncbi.nlm.nih.gov/geo/query/acc.cgi?acc=GSE33113 (http://www.ncbi.nlm.nih.gov/geo/query/acc.cgi?acc=GSE33113); http://www.ncbi.nlm.nih.gov/geo/query/acc.cgi?acc=GSE37892 (http://www.ncbi.nlm.nih.gov/geo/query/acc.cgi?acc=GSE37892); http://www.ncbi.nlm.nih.gov/geo/query/acc.cgi?acc=GSE38832 (http://www.ncbi.nlm.nih.gov/geo/query/acc.cgi?acc=GSE38832); http://www.ncbi.nlm.nih.gov/geo/query/acc.cgi?acc=GSE39582 (http://www.ncbi.nlm.nih.gov/geo/query/acc.cgi?acc=GSE39582); http://www.ncbi.nlm.nih.gov/geo/query/acc.cgi?acc=GSE62254 (http://www.ncbi.nlm.nih.gov/geo/query/acc.cgi?acc=GSE62254).
