# Supplementary figures and images for: A stroma‐related lncRNA panel for predicting recurrence and adjuvant chemotherapy benefit in patients with early‐stage colon cancer
Source: J Cell Mol Med. 2020 Jan 27;24(5):3229–41. doi: 10.1111/jcmm.14999 (PMC7077592; doi:10.1111/jcmm.14999)

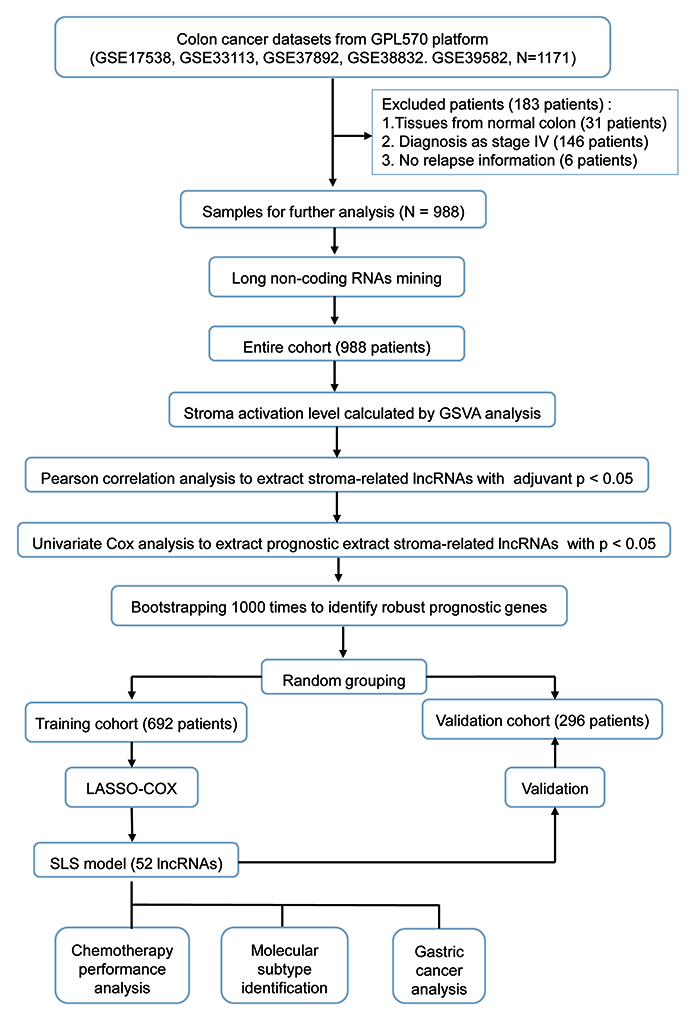

Supplement: Supplementary file 1 [file JCMM-24-3229-s001.tif]

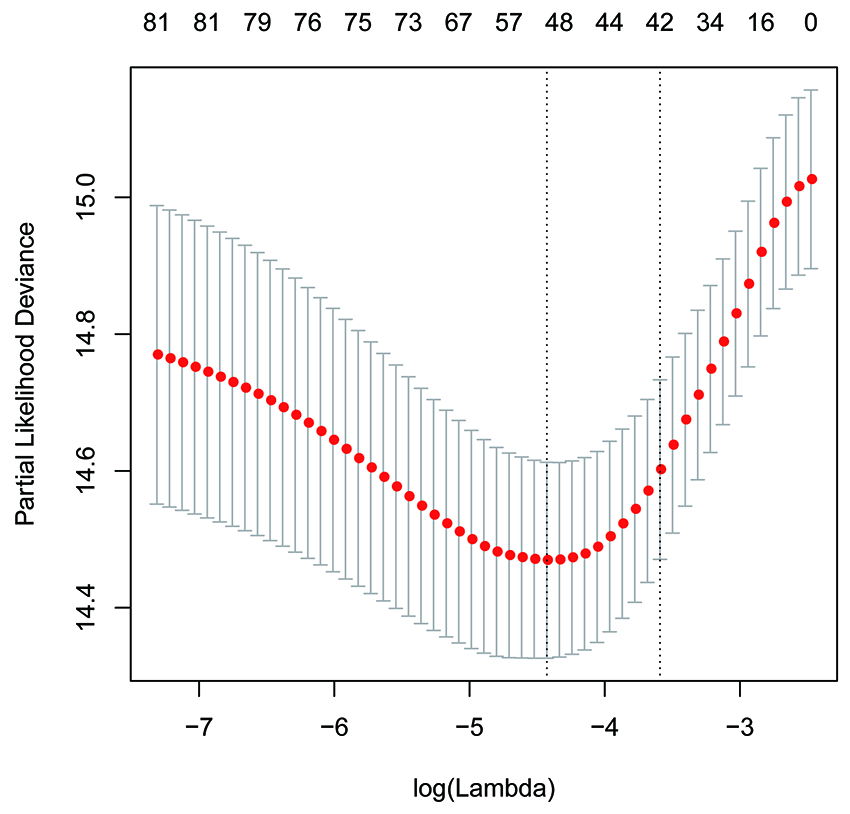

Supplement: Supplementary file 2 [file JCMM-24-3229-s002.tif]
