# Supplementary material for: A stroma‐related lncRNA panel for predicting recurrence and adjuvant chemotherapy benefit in patients with early‐stage colon cancer
Source: J Cell Mol Med. 2020 Jan 27;24(5):3229–41. doi: 10.1111/jcmm.14999 (PMC7077592; doi:10.1111/jcmm.14999)
Supplement: Supplementary file 4 [file JCMM-24-3229-s004.docx]

**Supplemental Table S1. Basic information of gene expression profiling series used in this study**

| **Datasets** | **Platform** | **Country** | **No. of patients** | **Cancer type** | **Analysis** |
| --- | --- | --- | --- | --- | --- |
| GSE17538 | GPL570 | USA | 176 | COAD | Model |
| GSE33113 | GPL570 | USA | 90 | COAD | Model |
| GSE37892 | GPL570 | France | 130 | COAD | Model |
| GSE38832 | GPL570 | USA | 92 | COAD | Model |
| GSE39582 | GPL570 | France | 500 | COAD | Model/Chemotherapy |
| GSE62254 | GPL570 | Korea | 222 | STAD | Model/Chemotherapy |

Abbreviation: *COAD, colon adenocarcinoma; STAD, stomach adenocarcinoma*
