# Supplementary material for: A stroma‐related lncRNA panel for predicting recurrence and adjuvant chemotherapy benefit in patients with early‐stage colon cancer
Source: J Cell Mol Med. 2020 Jan 27;24(5):3229–41. doi: 10.1111/jcmm.14999 (PMC7077592; doi:10.1111/jcmm.14999)
Supplement: Supplementary file 6 [file JCMM-24-3229-s006.docx]

**Supplemental Table S3. Patients’ basic characteristics**

| **Item** | **Entire cohort** | **Random grouping** | | |
| --- | --- | --- | --- | --- |
|  |  | **Training cohort** | **Validation cohort** | **P** |
| **Age**  (mean ± SD) | 67.48 ± 13.13 | 67.46 ± 12.69 | 67.46 ± 14.16 | 1.00 |
| **Gender** (n, %) |  |  |  | 0.88 |
| Male | 481 (53.1) | 333 (48.1) | 147 (49.7) |  |
| Female | 417 (42.1) | 293 (42.3) | 123 (41.6) |  |
| Unknown | 92 (9.3) | 66 (9.5) | 26 (8.8) |  |
| **Stage** (n, %) |  |  |  | 0.91 |
| I | 82 (8.3) | 59 (8.5) | 23 (7.8) |  |
| II | 532 (53.7) | 373 (53.9) | 159 (53.7) |  |
| III | 376 (38.0) | 260 (37.6) | 114 (38.5) |  |
| **Tumor site**  (n, %) |  |  |  | 0.33 |
| Proximal | 291 (29.4) | 194 (28.0) | 97 (32.8) |  |
| Distal | 198 (20.0) | 141 (20.4) | 57 (19.3) |  |
| Unknown | 501 (50.6) | 357 (51.6) | 142 (48.0) |  |
| **CMS** (n, %) |  |  |  | 0.54 |
| CMS1 | 148 (14.9) | 106 (15.3) | 42 (14.2) |  |
| CMS2 | 305 (30.8) | 217 (31.4) | 88 (29.6) |  |
| CMS3 | 103 (10.4) | 68 (9.8) | 35 (11.8) |  |
| CMS4 | 157 (15.9) | 103 (14.9) | 54 (18.2) |  |
| Unknown | 277 (28.0) | 198 (2.6) | 83 (26.0) |  |
| Abbreviation: *SD, standard deviation; CMS, consensus molecular subtypes* | | | |  |
