# Supplementary material for: A stroma‐related lncRNA panel for predicting recurrence and adjuvant chemotherapy benefit in patients with early‐stage colon cancer
Source: J Cell Mol Med. 2020 Jan 27;24(5):3229–41. doi: 10.1111/jcmm.14999 (PMC7077592; doi:10.1111/jcmm.14999)
Supplement: Supplementary file 8 [file JCMM-24-3229-s008.docx]

**Supplemental Table S5. Multivariate survival analyses of SLS model and clinical variables in the GSE39582 series**

| **Item** | **MVA for RFS** | | | **MVA for OS** | | |
| --- | --- | --- | --- | --- | --- | --- |
|  | **HR (95% CI)** | | **P value** | **HR (95% CI)** | | **P value** |
| Age^a^ | NE | | | 1.05 (1.03-1.07) | | < 0.001 |
| Gender (vs. male) | NE | | | 0.58 (0.40-0.85) | 0.005 | |
| SLS^a^ | 1.50 (1.36-1.66) | < 0.001 | | 1.47 (1.27-1.70) | < 0.001 | |
| Tumor site  (vs. proximal) | 1.56 (1.07-2.27) | 0.021 | | NE | | |
| Stage (vs. stage I) |  |  | | NE | | |
| Stage II | 5.15 (0.71-37.32) | 0.105 | |  | | |
| Stage III | 7.28 (1.00-52.90) | 0.050 | |  | | |
| Chemo  (vs. unconducted | NE | | | NE | | |
| C.subtype  (vs. C1) | NE | | |  | |  |
| C2 |  |  |  | 0.66 (0.38-1.16) | | 0.148 |
| C3 |  |  |  | 0.40 (0.18-0.87) | | 0.022 |
| C4 |  |  |  | 1.14 (0.63-2.05) | | 0.674 |
| C5 |  |  |  | 0.53 (0.32-0.88) | | 0.013 |
| C6 |  |  |  | 0.62 (0.31-1.23) | | 0.170 |
| CMS subtype  (vs. CMS4) | NE | | | NE | | |
| CMS1 |  |  |  |  |  |  |
| CMS2 |  |  |  |  |  |  |
| CMS3 |  |  |  |  |  |  |
| ^a^ Continuous variable  Abbreviation: *SLS, stroma-related LncRNA signature; RFS, relapse-free survival; OS, overall survival; MVA, multivariate analysis; HR, hazard ratio; CI, confidence interval; CMS, consensus molecular subtypes; NE, not enter.* | | | | | | |
