# Supplementary material for: A stroma‐related lncRNA panel for predicting recurrence and adjuvant chemotherapy benefit in patients with early‐stage colon cancer
Source: J Cell Mol Med. 2020 Jan 27;24(5):3229–41. doi: 10.1111/jcmm.14999 (PMC7077592; doi:10.1111/jcmm.14999)
Supplement: Supplementary file 9 [file JCMM-24-3229-s009.docx]

**Supplemental Table S6. Patients’ basic characteristics of GSE62254 dataset**

| **Item** | **Entire cohort** |
| --- | --- |
| **Age** (mean ± SD) | 62.42±10.74 |
| **Gender** (n, %) |  |
| Male | 157 (70.7) |
| Female | 65 (29.3) |
| **Stage** (n, %) |  |
| I | 30 (13.5) |
| II | 96 (43.2) |
| III | 96 (43.2) |
| **Lauren** (n, %) |  |
| Intestinal | 121 (54.5) |
| Diffuse | 93 (41.9) |
| Mixed | 8 (3.6) |
| **ACRG subtype** |  |
| EMT | 27 (12.2) |
| MSI | 59 (26.6) |
| MSS/TP53- | 75 (33.8) |
| MSS/TP53+ | 61 (27.5) |

Abbreviation: *ACRG, Asian Cancer Research Group; EMT, epithelial-to-mesenchymal transition; MSI, microsatellite instability; MSS, microsatellite stable*
