# Supplementary material for: A stroma‐related lncRNA panel for predicting recurrence and adjuvant chemotherapy benefit in patients with early‐stage colon cancer
Source: J Cell Mol Med. 2020 Jan 27;24(5):3229–41. doi: 10.1111/jcmm.14999 (PMC7077592; doi:10.1111/jcmm.14999)
Supplement: Supplementary file 10 [file JCMM-24-3229-s010.docx]

**Supplemental Table S7. Multivariate survival analyses of SLS model and clinical variables in the GSE62254 series**

| **Item** | **HR (95% CI)** | **p-value** |
| --- | --- | --- |
| Age^a^ | NE | |
| Gender (vs. male) | NE | |
| SLS | 2.69 (2.15-3.36) | < 0.001 |
| Lauren  (vs. Intestinal) | NE | |
| Stage (vs. stage I) | NE | |
| Stage II |  |  |
| Stage III |  |  |
| Chemo  (vs. unconducted) | 1.68 (1.04-2.71) | 0.033 |
| ACRG subtype  (vs. EMT) | NE | |
| MSI |  |  |
| MSS/TP53- |  |  |
| MSS/TP53+ |  |  |

^a^ Continuous variable

Abbreviation: *HR, hazard ratio; CI, confidence interval; ACRG, Asian Cancer Research Group; EMT, epithelial-to-mesenchymal transition; MSI, microsatellite instability; MSS, microsatellite stable; NE, not enter.*
